# Targeting of the AKT/m-TOR Pathway: Biomarkers of Resistance to Cancer Therapy——AKT/m-TOR Pathway and Resistance to Cancer Therapy

**DOI:** 10.3779/j.issn.1009-3419.2018.01.09

**Published:** 2018-01-20

**Authors:** Liudmila V SPIRINA, Irina V KONDAKOVA, Natalia V TARASENKO, Elena M SLONIMSKAYA, Evgeny A USYNIN, Alexey K GORBUNOV, Zahar A YURMAZOV, Svetlana Yu CHIGEVSKAYA

**Affiliations:** 1 Laboratory of Tumor Biochemistry, Cancer Research Institute, Tomsk National Research Medical Center of the Russian Academy of Sciences, Russia; 2 Department of Biochemistry and Molecular Biology, Siberian State Medical University, Tomsk, Russia; 3 Department of Medical Genetics, Siberian State Medical University, Tomsk, Russia; 4 Laboratory of Population Genetics, Research Institute of Medical Genetics, Tomsk National Research Medical Center of the Russian Academy of Sciences, Russia; 5 Surgical Department, Cancer Research Institute, Tomsk National Research Medical Center of the Russian Academy of Sciences, Russia; 6 Department of Oncology, Siberian State Medical University, Tomsk, Russia

**Keywords:** AKT/m-TOR signaling pathway, Resistance to cancer therapy, Molecular markers

## Abstract

Resistance to cancer therapy continues to be a major limitation for the successful treatment of cancer. There are many published studies on therapy resistance in breast and prostate cancers; however, there are currently no data on molecular markers associated with resistance. The conflicting data were reported regarding the AKT/m-TOR signaling pathway components as markers predicting resistance. The AKT/m-TOR signaling pathway is involved in the development of many human cancers; its activation is related to cell proliferation, angiogenesis, apoptosis, as well as to therapy resistance. Molecular alterations in the AKT/m-TOR signaling pathway provide a platform to identify universal markers associated with the development of resistance to cancer therapy.

## Introduction

The PI3K/AKT/mTOR pathway is a well established driver of cancer in humans^[1]^. Phosphatidylinositide 3 kinases (PI3Ks), AKT and mTOR constitute the core components of the PI3K/AKT/mTOR signaling cascade, which is important in regulating the cell cycle. Therefore, it is directly related to cellular quiescence, proliferation, apoptosis and cancer^[2]^. PI3K activation phosphorylates and activates AKT, localizing it in the plasma membrane. AKT phosphorylation is regulated by PDK1 (pyruvate dehydrogenase kinase) and mTORC2 complex. Currently it is known that mTORC2 consists of m-TOR, GβL, rictor (rapamycin-insensitive companion of TOR), mSin1 [mammalian stress-activated protein kinase (SAPK)-interacting protein 1] and protor (protein observed with rictor)^[3]^.

Proteins participating in cell growth, proliferation and apoptosis are the AKT substrates. Among them, there are c-RAF (serine/threonine-protein kinase) and GSK-3-beta (glycogen synthase kinase-3-beta)^[4]^. AKT indirectly activates the mTORC1 complex, which consists of m-TOR, raptor (regulatory-associated protein of TOR), mLST8 (mammalian lethal with Sec13 protein 8), or GβL, and PRAS40 (proline-rich PKB/AKT substrate 40 kDa). The mTOR protein forms at least two distinct multi-protein complexes, complex 1 (mTORC1) and complex 2 (mTORC2)^[5]^. The activated mTORC1 phosphorylates downstream effectors, including serine/threonine kinase p70S6K1 (S6K1) and 4EBP1^[2, 6]^. The targets of S6K1 include ribosomal proteins and elongation factors. The 4EBP1 inhibits the initiation of protein translation.

The negative feedback from the PTEN tumor suppressor phosphatase remains the most important factor regulating AKT activity^[7]^. PTEN specifically catalyses the dephosphorylation of the 3' phosphate of the inositol ring in PIP3, resulting in the biphosphate product PIP2. This dephosphorylation is important because it results in inhibition of the AKT signaling pathway. PH domain and Leucine rich repeat Protein Phosphatase (PHLPP) is also a PTEN substrate. PHLPP dephosphorylates Ser-473 (the hydrophobic motif) in AKT, thus partially inactivating the kinase. PHLPP may act as a tumor suppressor in several types of cancer due to its ability to block growth factor-induced signaling in cancer cells^[8]^.

The AKT-mTOR pathway is necessary to promote growth and proliferation over differentiation of adult cells and to inhibit apoptosis. This pathway is frequently hyperactivated in cancer cells and may also influence disease course and outcomes. It is known that 35% of breast cancer patients have a *PI3K* mutation. This genetic alteration is also present in 50%-70% of patients with non-small cell lung carcinoma, renal cell carcinoma, prostate cancer, colorectal cancer and etc. That is frequently associated with the loss of PTEN tumor suppressor^[9, 10]^. The modified expression of AKT/m-TOR signaling pathway components can influence the development of resistance to cancer therapies, which in turn results in poor prognosis.

## Resistance to cancer therapy and AKT/m-TOR signaling cascade

The development of resistance to cancer agents is the major reason for failure in cancer therapy. Clinically, cancer resistance can arise prior to or as a result of cancer therapy. There is evidence that "primary" or "*de novo*" resistance is a genetically determined event. Moreover, nearly all patients having initial tumor response inevitably become refractory to the therapy ("secondary" or "acquired" resistance). There are many published studies on resistance to endocrine therapy for breast and prostate cancers. Most patients with breast cancer are known to have hormone receptor-positive (HR+) tumors. HR+ breast cancers generally have a favorable prognosis^[11]^. However, despite advances in the treatment of HR+ tumors, approximately 30% of these patients will eventually experience relapse with metastatic disease^[12]^. Therapy with androgen deprivation therapy (ADT) benefits over 80% of patients with locally advanced prostate cancer, but the remaining patients ultimately develop progressive disease resulting in castrate-resistant prostate cancer^[13, 14]^.

Intrinsic or acquired resistance is a major limitation of targeted cancer therapies. Targeted therapy for metastatic renal cell carcinoma was found to increase the time to progression from 5 to 12 months and the overall survival from 12 to 24 months, with the objective response rate of 40%^[9, 15-18]^. However, the response rate in patients with metastatic renal cell carcinoma, who did not receive targeted therapy, was approximately 5%. Despite the generally good prognosis of thyroid carcinoma, about 5%-15% of patients will develop metastatic disease which fails to respond to radioactive iodine, exhibiting a more aggressive behavior.

Different approaches used to discover markers for predicting cancer drug resistance are being currently developed. The AKT/m-TOR signaling pathway is a promising therapeutic target that has been well established to play a very significant role in tumor cell growth and proliferation^[19, 20]^.

The AKT/m-TOR pathway activity is associated with resistance to cancer therapy. Changes in the AKT/m-TOR pathway activity can result in the development of castrate-resistant prostate cancer^[21]^. Ineffective ADT for prostate cancer is associated with decreased activity of the AKT/m-TOR pathway. The switch of AKT/m-TOR cascade on MAPK and JAK/STAT signaling pathways is pivotal in prostate cancer prognosis^[22]^. The application of novel AKT inhibitors offers the potential of blocking castrate-resistant prostate cancer cell growth and survival^[23]^. The biological behavior of cancer is involved in the development of primary and acquired resistance to targeted therapy in kidney cancer patients^[24]^. One third of these patients are inherently resistant to the targeted agents^[25]^. Hyperactivation of AKT/m-TOR signaling pathway is observed in kidney cancer patients who failed to respond to tyrosine kinase inhibitors. There is evidence that the AKT/m-TOR signaling pathway components may be perspective markers predicting the development of resistance to targeted therapy. The VEGF, HIF, AKT and m-TOR are known to be potential markers for predicting resistance to cancer therapy; however their significance is still unclear^[26]^. The HIF-1, VEGF or TORC2 overexpression in a case of m-TOR inhibition leads to increase in PI3K and AKT activities^[27, 28]^.

The PI3K has been associated with resistance to endocrine therapy, human epidermal growth factor receptor 2 (HER2)-directed therapy and cytotoxic therapy in breast cancer^[29]^. PI3K has independently been implicated in trastuzumab resistance. Multiple inhibitors of the AKT/m-TOR pathway are in preclinical development or are already in clinical trials. There are promising data indicating that rapalogs or inhibitors of PI3K/AKT are active in breast cancers^[30]^.

The combination of dual PI3K/AKT/m-TOR inhibitors (BEZ235 or PI103) with radiotherapy is a promising modality for the treatment of castrate-resistant prostate cancer to overcome radioresistance^[31]^. A crucial role for angiogenesis inhibitors in shifting the fate of radiation-induced HIF-1alpha activity from hypoxia-induced tumor radioresistance to hypoxia-induced tumor apoptosis was found^[32]^. Current data highlight the potential role of AKT/m-TOR signaling in thyroid carcinoma progression^[33]^. The m-TOR signaling complex was also found to be associated with activated AKT and 4E-BP1 in thyroid cancers^[34]^. Studies of Lin *et al*. indicated the PI3-kinase activation by thyroid hormones^[33, 35]^. A prominent role of PI3K and HIF-1 signaling in metastatic papillary and follicular thyroid cancer was established^[33, 36, 37]^. Moreover, the activation of AKT/m-TOR signaling pathway was correlated with poor response to chemotherapy with cisplatin^[20, 21, 38]^.

## Conclusion

Thus, there are numerous data on the relationship between the response to cancer therapy and activation of the AKT/m-TOR signaling pathway. In fact, the role of molecular mechanisms in the development of resistance to cancer therapy remains unclear. In conclusion, it should be noted that the study of AKT/m-TOR signaling cascade is a potential platform for identifying effective markers associated with the development of resistance to cancer therapy. This statement is related to the universality of AKT/m-TOR signaling pathway playing a significant role in cell proliferation and apoptosis. The modified activity of AKT/m-TOR signaling pathway underlies the molecular mechanism of cancer development and implicates in the resistance to cancer therapy.

## Conflict of interest statement

Authors declare that they have no conflict of interest
